# Counting ECGs in acute psychiatry – The patients' price for junior doctors' rotations

**DOI:** 10.1192/bjo.2021.301

**Published:** 2021-06-18

**Authors:** Marianna Rogowska, Adam Montgomery, Luiz Dratcu

**Affiliations:** John Dickson Ward, Maudsley Hospital

## Abstract

**Aims:**

On 05/08/20, when a new cohort of doctors rotated onto an acute ward, (John Dickson Ward, Maudsley Hospital, London) a new handover tool on MS Teams was introduced, which replaced previously used MS Word document. The new handover tool can be accessed and edited by any of the users in the team. We hypothesised that the introduction of an interactive, live-updated tool would help improve physical health monitoring for patients, especially compliance with ECG taking. The aim of this project was to test this hypothesis.

**Method:**

Authors have reviewed electronic documentation of patients admitted to and discharged from John Dickson Ward between 01/04/2020 and 24/12/2020. Evidence of whether an ECG was performed, was offered but declined by the patient, or was not offered were noted in the final audit. Patients were divided into 3 groups: (1) Patients admitted and discharged from 01/04/20 – 05/08/2020; (2) Patients admitted and discharged from 05/08/2020 – 24/12/20, and (3) Patients admitted before the intervention date, but discharged after the date (i.e., the period when new junior doctors had rotated onto the ward). Fifty patient records were identified in Group 1, fifty in Group 2, and 18 in Group 3.

**Result:**

Surprisingly, the percentage of patients who had a documented ECG did not improve after the intervention, with 37/50 (74%) of patients having an ECG in Group 1, and 37/50 (74%) of patients having an ECG in Group 2. However, an incidental finding was made that significantly fewer patients received ECGs during the changeover period (Group 3), with only 6/18 (33%) of patients receiving ECGs. The percentage of patients who were not offered ECGs also increased during the changeover period, with 2/50 (4%) in Group 1, and 3/18 (17%) in Group 3 not being offered.

**Conclusion:**

This incidental finding highlights the challenges associated with the junior doctor changeover period. Much time is needed for doctors to adjust to their new surroundings and methods of working, and this may result in basic elements of patient care being overlooked. We surmise that other elements, such as ensuring all patients having regular blood tests and physical examinations, may also be of a lower standard during this period. There is scope for future audits to address this, and for future quality improvement projects to implement changes ensuring medical care remains at a high standard during junior doctor changeover periods.

